# Evaluation of statistical illiteracy in Latin American clinicians and the piloting evaluation of a short course across multiple timepoints

**DOI:** 10.1186/s12909-022-03128-w

**Published:** 2022-01-25

**Authors:** Adrian Soto-Mota, Eduardo Carrillo Maravilla, Jose Luis Cárdenas Fragoso, Óscar Arturo Lozano Cruz, Alfonso Gulías Herrero, Sergio Ponce De Leon Rosales

**Affiliations:** 1 Metabolic Diseases Research Unit, National Intitute of Medical Sciences and Nutrition Salvador Zubiran, Mexico City, Mexico; 2 Medical Division at The National Institute of Medical Sciences and Nutrition Salvador Zubirán, Mexico City, Mexico

**Keywords:** Statistical illiteracy, Evidence-based medicine, Medical education

## Abstract

**Background:**

All clinicians require statistical interpretation skills to keep up to date with evidence-based recommendations in their field. However, statistical illiteracy among clinicians is a highly prevalent problem with far-reaching consequences. The few available that report statistical literacy improvements after educational interventions do not measure for how long these benefits last. To estimate statistical illiteracy among Latin-American clinicians across multiple levels of training and to evaluate a 10-h course at multiple timepoints.

**Methods:**

Using an online questionnaire, we evaluated; self-perceived statistical proficiency, scientific literature reading habits and statistical literacy (using an adaptation of the Quick Risk Test). Separately, we evaluated statistical proficiency after a 10-h statistics course in a group of Internal Medicine residents at a tertiary center in Mexico City across multiple time points between November 2020 and February 2021.

**Results:**

Data from 392 clinicians from 9 Latin American countries were analyzed. Most clinicians (85%) failed our adaptation of the Quick Risk Test (mean score = 2.6/10, IQR:1.4). The 10-h course significantly improved the scores of the Internal Medicine Residents (*n* = 16) from 3.8/10, IQR:1.8 to 8.3/10, IQR:1.4 (*p* < 0.01). However, scores dropped after one and 2 months to 7.7/10, IQR:1.6 and 6.1 / 10, IQR:2.2, respectively.

**Conclusion:**

Statistical Illiteracy is highly prevalent among Latin American clinicians. Short-term educational interventions are effective but, their benefits quickly fade away. Medical boards and medical schools need to periodically teach and evaluate statistical proficiency to ameliorate these issues.

## Introduction

Most medical schools and medical board recognise the importance of Statistical Skills for practising clinicians [1]. However, evidence shows that even experienced clinicians struggle with assimilating the differences and implications of fundamental statistical concepts such as odds ratio versus absolute risk and sensitivity versus positive post-test probability [2]. Moreover, essential concepts such as absolute risk changes, number needed to treat/screen, intention-to-treat analysis and Bayesian probability are often overlooked when making clinical decisions and when explaining the implications of tests and treatments to patients [3, 4].

The implications of Statistical Illiteracy among clinicians are frequent and range from generating individual ethical problems [5–7] to health-policy misinformed decisions [8]. Moreover, improving health statistics among medical doctors has been put forward as one of the seven goals for improving health during this century [9].

Importantly, evidence also suggests that cheap, easy-to-implement and short-term interventions can improve statistical skills among clinicians [10]. In their 2018 study, Jenny, Keller and Gigerenzer [11] demonstrated that a 90-min training session in medical statistical literacy improved the performance (from 50 to 90%) in 82% of the participants using a multiple-choice Statistics test. However, it was not evaluated how quickly these improvements fade away after the educational intervention. In this study, we estimated Statistical Literacy among Latin American clinicians and evaluated the efficacy of a 10-h Statistics course across multiple timepoints.

## Methods

### Online survey and statistics test

An online survey collected information about medical training, medical school and graduation year, self-perceived understanding of the methods section of scientific papers (as a percentage), and the number of scientific papers read per week, and extracurricular statistical training. Email restrictions were placed to ensure respondents were only capable of answering once. The survey it can be reviewed at https://forms.gle/fCep4atAhcoG5BKW6

The test was based on the Quick Risk Test [11] but, to avoid granting points by guessing, was modified to incorporate an “I don’t know” option in all questions. Additionally, it avoided word by word translations and evaluating concepts by directly asking their definitions. Hypothetical cases and examples were used instead. The evaluated concepts were: Sensitivity, Specificity, Positive and Negative Predictive Values, Statistical Power, Sample Size, Statistical Significance, Statistical correlation, Absolute and Relative Risk, Bayesian reasoning and Dependent and Independent probabilities. Respondents did not receive feedback after each question to avoid their early performance influenced their final answers.

### Characteristics and design of the educational intervention

A 10-h course was divided into ten one-hour weekly sessions to review each one of the concepts evaluated by the test. All sessions were recorded and available for review during the 10 weeks the course lasted.

This course was summarized into a 3-h long 10 session videos now freely available at: https://www.youtube.com/watch?v=cdEX8AdEU6Y&list=PLoieIsf7siGMTkICPbkvgD1hyZpbVwd0H

### Evaluation of the efficacy of the course

Due to the time available in their academic program, Internal Medicine residents at a tertiary center in Mexico answered the test before the course, immediately after the last lecture, 1 month after the course, and 2 months after the course (between November 2020 and February 2021). This group was totally independent and separately recruited from the group that answered the online survey without a specific sampling procedure. Residents were invited to voluntarily attend the lectures and answer the tests.

Lecture recordings were unavailable after the course ended to avoid biasing the follow-up evaluations.

The same questions were used for all evaluations except for the very last one in which, different cases evaluated the same Statistical concepts.

### Statistical analysis

Since scores were not normally distributed, we compared them using Friedman’s Test using the R function “friedman.test” from the R package “stats” version 3.6.2. Normality was evaluated using Shapiro-Wilk tests using the function “shapiro.test” in the same R package.

## Results

### Survey responses and statistical literacy results among Latin American clinicians

**Fig. 1:**
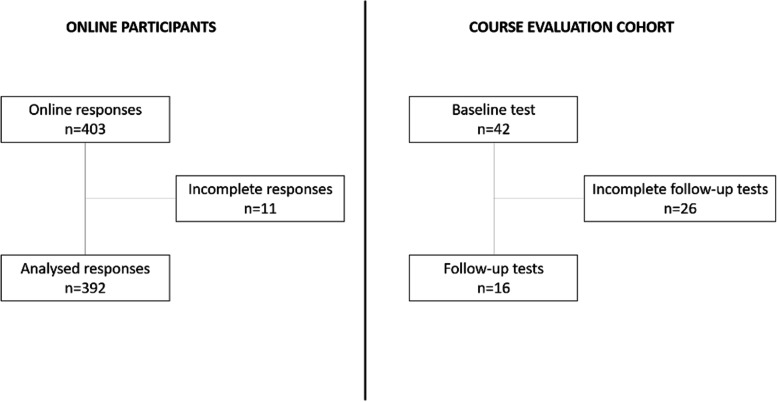
Participants workflow for the online survey and for the piloting of a 10 short course. The online survey was distributed using social media. Clinicians who did not answer all tests were excluded from the final analysis

A total of 403 responses were collected, however, 11 were discarded due to having incomplete data. Figure 1 describes our study’s participant workflow. In total 392 from 9 different countries and 53 different medical schools were included in the analysis. Table 1 describes their educational background characteristics. Table 2 summarizes tests results across different levels of medical training, scores were not significantly different (*p* > 0.05). Table 3 describes the percentage of correct answers in every statistical concept we evaluated.

**Table 1:** Participants’ educational background and reading habits

Educational background	Frequency (percentage)
Medical training level	
Undergraduate	103 (26.3%)
General Practitioner	102 (26.0%)
Resident	44 (11.3%)
Graduated Specialist	142 (36.4%)
Extra-curricular statistical training	82 (21%)
Years after medical graduation	Mean: 6, std. dev.: +/− 3
Declared number of scientific papers read per week	Mean: 3, std. dev.: +/− 2
Self-perceived level of Methodological understanding.	Mean:55/100, std. dev.: +/− 17
Mexican	321 (81.8%)
Argentinian	19 (4.8%)
Colombian	15 (3.8%)
Peruvian	15 (3.9%)
Other countries	19 (4.8%)

*n* = 392

**Table 2:** Test results across different levels of medical training

Test scores by medical training level	Test result
Undergraduate	2.7 (IQR: 1.2 – 3.8)
General Practitioner	2.2 (IQR: 1.5 – 4.0)
Resident	3.4 (IQR: 1.4 – 4.1)
Evaluation group (residents)	3.8 (IQR: 2.9 – 4.2)
Graduated Specialist	2.9 (IQR: 1.9 – 3.9)
Overall tests score	2.6 (IQR: 1.5 – 3.3)

*n* = 392. Test’s scale is 0 – 10. Data are medians and IQR = interquartile range. Groups’ results were not statistically different when compared with a Friedman’s test (*p* > 0.05)

**Table 3:** Baseline performance in every evaluated concept

Statistical concept	Proportion of correct answers (online / ev. group)	Admitting “I don’t know.” (online / ev. group.)
Sensitivity and Specificity	94 (23.4%) / 4 (25.0%)	43 (10.9%) / 5 (31.2%)
Positive & Negative Predictive Values	246 (62.7%) & 262 (67%) / 4 (25.0%) & 5 (31.2%)	11 (2.8%) & 156 (39.7%)/ / 2 (12.5%) & 7 (43.7%)
Statistical Power	125 (31.88%) / 5 (31.2%)	62 (15.8%) / 9 (56.2%)
Sample Size	74 (18.87%) / 3 (18.7%)	39 (9.9%) / 9 (56.2%)
Statistical Significance	145 (36.9%) / 6 (37.5%)	121 (30.8%) / 9 (56.2%)
Absolute and Relative Risk	70 (17.8%) / 4 (25.0%)	82 (20.9%) / 5 (31.2%)
Bayesian reasoning	174 (44.8%) / 7 (43.7%)	19 (4.8%) / 7 (43.7%)
Statistical correlation	121 (30.8%) / 5 (31.2%)	101 (25.7%) / 5 (31.2%)
Dependent & Independent probabilities	54 (13.7%) / 2 (12.5%)	19 (4.8%) / 6 (37.5%)

*ev. group* Evaluation group (Internal Medicine residents)

### Evaluation of the efficacy of the course

Internal Medicine residents at the National Institute of Medical Sciences and Nutrition Salvador Zubirán voluntarily attended or listened to the recorded lectures at their own pace and answered the Statistics tests at the already mentioned time points. Tests were self-paced, unsupervised and were open for 5 days during each time point. Email restrictions were placed to allow no more than one answer per resident.

**Fig. 2:**
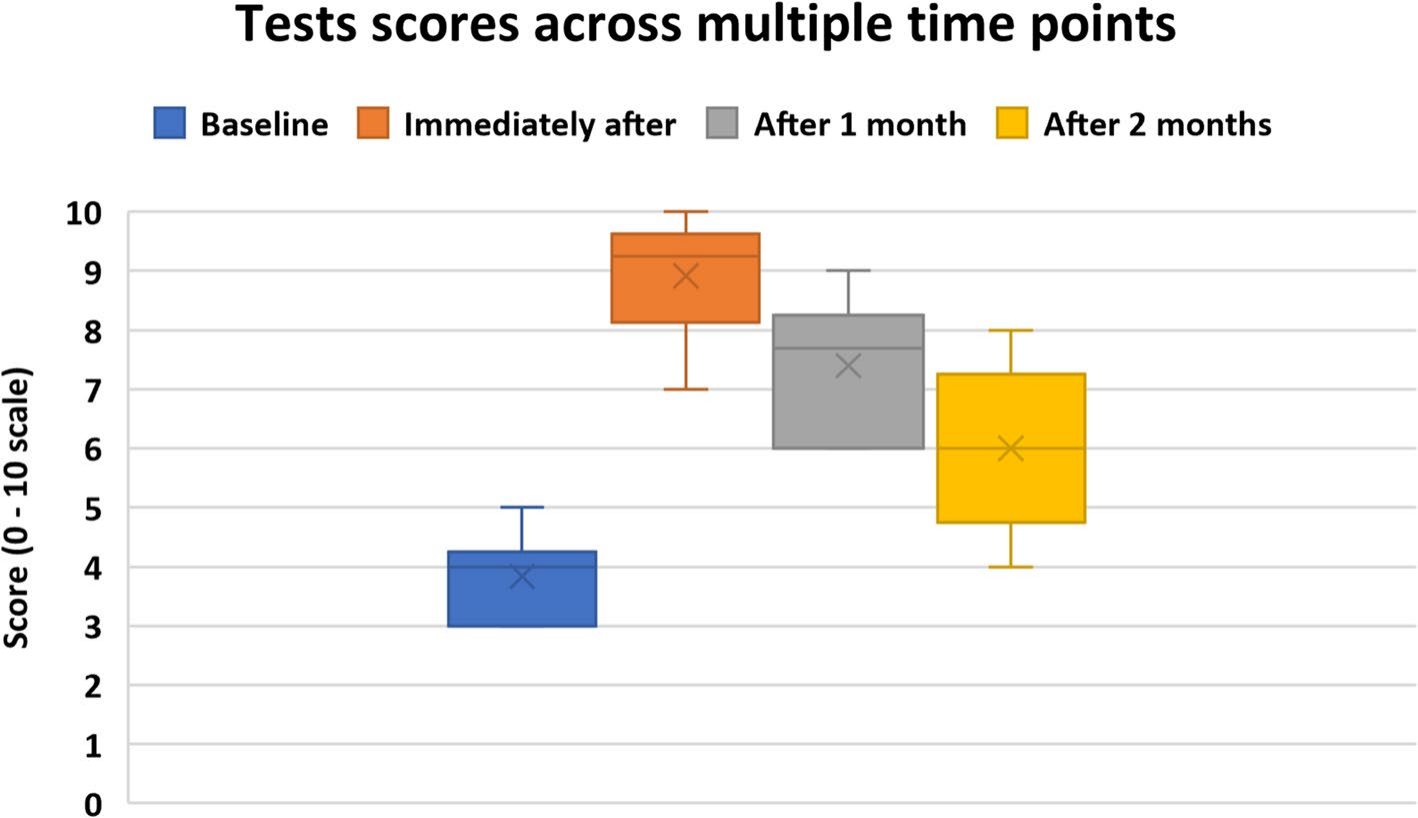
Test scores across multiple time-points. Boxplots represent medians and IQR = interquartile range. Groups’ results statistically different at all timepoints when compared with a Friedman’s test (*p* > 0.05). *n* = 16

Only those who answered all tests (*n* = 16 out of 42 residents) were included in the analysis. Figure 2 shows the scores results and their distributions across the evaluated time points. Timepoints were statistically different from baseline when compared with Friedman’s Test (*p* < 0.01). Further, follow up was stopped because the resident’s academic year ended in March 2021. Table 4 summarizes the correct answers in every evaluated concept immediately after and 2 months after the 10-h course.

**Table 4:** Correct answers in every evaluated concept immediately after and two months after the 10-h course

Statistical concept	Immediately after the 10-h course	Two months after the 10-h course
Sensitivity and Specificity	15 (93.7%)	11 (68.7%)
Positive & Negative Predictive Values	14 (87.5%) & 13 (81.2%)	10 (62.5%) & 8 (50.0%)
Statistical Power	12 (75.0%)	9 (56.2%)
Sample Size	14 (87.5%)	6 (37.5%)
Statistical Significance	11 (68.7%)	7 (43.7%)
Absolute and Relative Risk	15 (93.7%)	9 (56.2%)
Bayesian reasoning	12 (75.0%)	8 (50.0%)
Statistical correlation	13 (81.2%)	11 (68.7%)
Dependent & Independent probabilities	12 (75.0%)	10 (62.5%)

## Discussion

To our knowledge, this is the first attempt at estimating Statistical illiteracy among Latin American Clinicians. Despite having found comparable statistical proficiency scores to those in other countries [11], this population merits being analysed separatedly because most educational tools that address this problem are only available in English [12, 13] and English proficiency is not mandatory for practicing Medicine in all Latin American countries.

In contrast with Jenny, Keller and Gigerenzer study [11], we allowed for respondents to admit they did not know an answer to the questions and measured for up to 2 months the lasting effects of the educational intervention we tried. The fact that scores quickly and significantly drop after a few weeks of having finished the course highlight the importance of continuous teaching and periodical evaluation. The finding that it is infrequent that clinicians recognise they do not know and the discrepancy between their self-perceived statistical skills and their tests scores suggest clinicians overestimate their statistical proficiency. Teaching clinicians to identify when they lack enough information or how to avoid cognitive biases should be emphasized when designing educational tools.

Some questions evaluated the same concepts by evaluating theoretical knowledge (i.e., Which of the following factors influences the positive predictive value of a test?) while others by presenting practical scenarios (i.e., How does the positive predictive value of an Influenza rapid test changes over the year?). Interestingly, clinicians performed better at theoretical questions (63% right answers) than with practical ones (15% right answers). Thus, it is likely than emphasizing practical interpretation over theoretical knowledge would yield better results when designing educational resources for improving statistical literacy.

The main limitations of our pilot study are inherent to the nature of self-reported, online-based studies. Also, Mexican clinicians were overrepresented in our sample and because we could control or confirm attendance with an open online course, our educational intervention was evaluated in a very small and highly specific group of clinicians (Mexican Internal Medicine residents). Nonetheless, the large number of participants and consistency of the results make it unlikely that more controlled methods would yield very different results.

In that regard, it is likely that, the reason behind the large discrepancies in the proportion of “I don’t know” answers between the online participants and the evaluation group are due to selection bias. The admission process for residency programs at the National Institute of Medical Sciences and Nutrition is highly competitive and includes a multiple-choice test where the only options are “True”, “False” and “I don’t know”. Correct answers are grant one point, errors deduced one point, and “I don’t know neither grant nor penalise points.

An additional limitation for extrapolating the utility of these short-term interventions comes from the fact there is not a consensus about which specific statistical skills are necessary for all physicians. Moreover, different types of specialists would likely require developing and preserving different skills. For example, clinical trials are more frequent in Internal Medicine Journals than in Forensic Medicine ones. Further research is needed.

Nonetheless, since it is not possible to practice Evidence-Based Medicine if clinicians cannot understand scientific evidence, further research is much needed to help guide future educational strategies and policies that help reduce the educational, ethical, and economic impact Statistical Illiteracy has on everyday medical practice.

## Conclusion

Similarly, to other populations, the group we evaluated also struggled with basic statistical concepts that are essential for correctly interpreting emerging evidence. Short-term educational interventions could improve statistical skills; however, these improvements seem to quickly fade away if they are not continuously reinforced. Our results highlight the need to periodically teach and evaluate statistical proficiency by medical schools and medical boards.

## Data Availability

Data for research purposes will be shared upon request to the corresponding author.

## References

[1] Johnson TV, Abbasi A, Schoenberg ED, Kellum R, Deann Speake L, Spiker C (2014). Numeracy among trainees: are we preparing physicians for evidence-based medicine?. J Surg Educ.

[2] Anderson BL, Williams S, Schulkin J (2013). Statistical literacy of obstetrics-Gynaecology residents. J Grad Med Educ.

[3] Naylor CD, Chen E, Strauss B (1992). Measured enthusiasm: does the method of reporting trial results alter perceptions of therapeutic effectiveness?. Ann Intern Med.

[4] Whiting PF, Davenport C, Jameson C, Burke M, Sterne JAC, Hyde C, et al. How well do health professionals interpret diagnostic information? A systematic review. BMJ Open. BMJ Publishing Group. 2015;5:1–8. 10.1136/bmjopen-2015-008155 PMC4521525 26220870

[5] Entwistle VA, Carter SM, Cribb A, McCaffery K (2010). Supporting patient autonomy: the importance of clinician-patient relationships. J Gen Intern Med.

[6] Sedrakyan A, Shih C (2007). Improving depiction of benefits and harms: analyses of studies of well-known therapeutics and review of high-impact medical journals. Med Care.

[7] Wegwarth O, Gigerenzer G (2018). The barrier to informed choice in cancer screening: statistical illiteracy in physicians and patients. Recent results in Cancer research.

[8] Iacobucci G (2018). Conservative conference: may announces new cancer strategy to boost survival rates. BMJ (Clinical research ed).

[9] Gigerenzer G, Gray JAM, Gigerenzer G, Gray JAM (2011). Launching the Century of the Patient. Better Doctors, Better Patients, Better Decisions.

[10] Garcia-Retamero R, Cokely ET, Wicki B, Joeris A (2016). Improving risk literacy in surgeons. Patient Educ Couns.

[11] Jenny MA, Keller N, Gigerenzer G (2018). Assessing minimal medical statistical literacy using the quick risk test: a prospective observational study in Germany. BMJ Open.

[12] Understanding Medical Research: Your Facebook Friend is Wrong | Coursera. Available from: https://www.coursera.org/learn/medical-research.

[13] Stanford Medical Statistics Certificate | Stanford Online. Available from: https://online.stanford.edu/programs/stanford-medical-statistics-certificate.

